# AI Deployment on GBM Diagnosis: A Novel Approach to Analyze Histopathological Images Using Image Feature-Based Analysis

**DOI:** 10.3390/cancers15205063

**Published:** 2023-10-19

**Authors:** Eva Y. W. Cheung, Ricky W. K. Wu, Albert S. M. Li, Ellie S. M. Chu

**Affiliations:** 1 School of Medical and Health Sciences, Tung Wah College, 31 Wylie Road, HoManTin, Hong Kong; albertli@twc.edu.hk; 2 Department of Biological and Biomedical Sciences, School of Health and Life Sciences, Glasgow Caledonian University, Glasgow G4 0BA, UK; ricky.wu@gcu.ac.uk; 3 Department of Clinical Pathology, Pamela Youde Nethersole Eastern Hospital, Hong Kong

**Keywords:** glioblastoma (GBM), primary brain tumor, artificial intelligence (AI), support vector machine (SVM), hematoxylin and eosin stained (H&E), histopathology, image features, GLCM, GLRLM, whole slide image

## Abstract

**Simple Summary:**

Glioblastoma (GBM) is one of the most common malignant primary brain tumors. The gold standard of cancer diagnosis relies on a medical technologist and a pathologist for feature-based analysis of hematoxylin and eosin-stained slides. To improve the efficiency of GBM diagnosis, an artificial intelligence model was built based on the TCGA-GBM dataset, and was deployed on an independent dataset collected locally. The support vector machine model showed excellent accuracy with very good sensitivity and specificity. It could potentially be used for GBM diagnosis and future clinical application.

**Abstract:**

Background: Glioblastoma (GBM) is one of the most common malignant primary brain tumors, which accounts for 60–70% of all gliomas. Conventional diagnosis and the decision of post-operation treatment plan for glioblastoma is mainly based on the feature-based qualitative analysis of hematoxylin and eosin-stained (H&E) histopathological slides by both an experienced medical technologist and a pathologist. The recent development of digital whole slide scanners makes AI-based histopathological image analysis feasible and helps to diagnose cancer by accurately counting cell types and/or quantitative analysis. However, the technology available for digital slide image analysis is still very limited. This study aimed to build an image feature-based computer model using histopathology whole slide images to differentiate patients with glioblastoma (GBM) from healthy control (HC). Method: Two independent cohorts of patients were used. The first cohort was composed of 262 GBM patients of the Cancer Genome Atlas Glioblastoma Multiform Collection (TCGA-GBM) dataset from the cancer imaging archive (TCIA) database. The second cohort was composed of 60 GBM patients collected from a local hospital. Also, a group of 60 participants with no known brain disease were collected. All the H&E slides were collected. Thirty-three image features (22 GLCM and 11 GLRLM) were retrieved from the tumor volume delineated by medical technologist on H&E slides. Five machine-learning algorithms including decision-tree (DT), extreme-boost (EB), support vector machine (SVM), random forest (RF), and linear model (LM) were used to build five models using the image features extracted from the first cohort of patients. Models built were deployed using the selected key image features for GBM diagnosis from the second cohort (local patients) as model testing, to identify and verify key image features for GBM diagnosis. Results: All five machine learning algorithms demonstrated excellent performance in GBM diagnosis and achieved an overall accuracy of 100% in the training and validation stage. A total of 12 GLCM and 3 GLRLM image features were identified and they showed a significant difference between the normal and the GBM image. However, only the SVM model maintained its excellent performance in the deployment of the models using the independent local cohort, with an accuracy of 93.5%, sensitivity of 86.95%, and specificity of 99.73%. Conclusion: In this study, we have identified 12 GLCM and 3 GLRLM image features which can aid the GBM diagnosis. Among the five models built, the SVM model proposed in this study demonstrated excellent accuracy with very good sensitivity and specificity. It could potentially be used for GBM diagnosis and future clinical application.

## 1. Introduction

Glioblastoma (GBM) is one of the most common malignant primary brain tumors, which accounts for 60–70% of all gliomas [1], and is classified as a grade IV tumor by the World Health Organization (WHO) based on histopathological features. Its invasiveness and high vascularity properties promote its spread throughout the brain parenchyma, resulting in a high frequency of recurrence and poor prognosis [2]. Early diagnosis and effective treatment planning is crucial in improving the survival rate of patients.

### 1.1. Morphology-Based Analysis

Compared to radiological imaging, which shows the location and the size of the tumor, histopathological images provide a more important basis for cancer diagnosis. Our previous studies illustrated that accurate identification of a tumor is crucial for treatment planning [3,4]. The gold standard of glioblastoma diagnosis and post-operation treatment planning is based on the identification of tumor cells on histopathological slides by both experienced medical technologists and pathologists. Morphological study of the cancer cells, including the shape, size, and structure of the components (first order statistics) within the cell and the cellular arrangement within the tissue will be investigated and compared to the normal cells and tissues. In view of cancer cells, they are characterized by their irregular size and shape, with a large nucleus, and with prominent nucleoli [5]. However, manual diagnosis of histopathological images requires pathologists who have been trained for years. Moreover, the diagnosis process is time consuming and labor intensive [6]. The development of AI-based histopathological image analysis further improves cancer diagnosis via accurate counting of cell types and/or quantitative analysis. However, due to the tumor heterogeneity, the accuracy of using the computer-assisted histopathological image analysis for GBM diagnosis still requires improvement [7].

### 1.2. Texture-Based Analysis

Recently, the advancement of digital pathology enables high-resolution histopathological image analysis, which allows researchers to access more features from computer-aided digital image analysis. It allows the presentation of detailed quantitative histo-morphometry features, including histologic primitives (e.g., glands and nuclei) from a standard hematoxylin and eosin (H&E) slide in the whole slide image [8]. A recent study applied the contouring of different cells from prostate cancer histopathology images to reduce the screening time for diagnosis and improve the diagnostic accuracy [9]. The high-resolution histopathological images also enable us to perform texture-based features analysis at pixel level, which could be a game-changer for cancer diagnosis. Gray level co-occurrence matrix (GLCM) and gray level run length matrix (GLRLM) are two categories of texture-based analysis methods. GLCM is the statistics of pixel intensity distribution. It considers the statistics of pairs of pixels in certain spatial relations to each other and provides information on the classification of textures [10]. GLRLM quantifies gray level runs, that is the length in number of pixels, with the same gray level value consecutively [11]. Both GLCM and GLRLM give a more comprehensive analysis of the histopathological image when compared to the traditional first order statistical analysis. Previous studies employed GLCM and GLRLM image features, mainly in radiological images for cancer screening [12], cancer diagnosis [13,14], prognosis prediction [15,16], and for disease types classification in dementia [17].

### 1.3. Machine Learning in Cancer Diagnosis

To handle a large number of image features in one pass effectively, machine learning is valued in analysis and diagnosis for medical images with minimal human intervention. The applications of machine learning include in medical imaging for organ segmentation [18,19], and co-registration of medical images from multiple modalities [20], so as to improve the accuracy in organs segmentation in CT images, and co-registration in multiple imaging modalities for accurate tumor localization. Furthermore, artificial intelligence algorithms can analyze medical images simultaneously with remarkable speed. It not only improves accuracy in handling large amount of medical and imaging data, but also significantly reduces the time required for diagnosis. Hence, computer-aided diagnosis in cancer using CT or MRI images has been proposed for lung cancer [21] and head and neck cancer diagnosis [22].

However, the application of machine learning in previous studies mainly focuses on radiological images. Recent studies applied machine learning to histopathological images analysis including breast cancer classification [23,24,25] and cervical cancer detection [26].

Therefore, in this study, we aimed to develop an AI system to aid pathologists in GBM diagnosis via texture-based image analysis. We employed texture-based analysis on the image features of histopathological slides, to identify the image features and their correlation to those features that pathologists used for the traditional diagnosis of GBM. Moreover, we further verified the AI system with local clinical data for future application.

## 2. Materials and Methods

### 2.1. Patient Dataset

There were two datasets involved in this study, The first dataset was the Cancer Genome Atlas Glioblastoma Multiforme Collection (TCGA-GBM), which was retrieved from The Cancer Imaging Archive (TCIA) [27]. There are 262 participants in this dataset, which come from eight oncology centers. This dataset comprises tissue slide images, CT images, MR images in DICOM format, as well as clinical data [28]. Clinical data, including the age at diagnosis, sex, tumor histology, overall staging, and survival days after confirmed diagnosis were also collected. The disease was staged by the 2007 World Health Organization (WHO) classification of tumors of the central nervous system [29].

The second dataset comprised hematoxylin and eosin (H&E) slide images collected from a local hospital in Hong Kong. Ethics approval was obtained from the IRB and was compliant with the guidance of Declaration of Helsinki, and the International Council for Harmonization of Technical Requirements for Pharmaceuticals for Human Use, Guideline for Good Clinical practice (ICH GCP) in order to safeguard the rights, safety, and well-being of research subjects. It comprised a group of 60 patients diagnosed with GBM during 2018–2020, and another group of 60 participants with no known brain diseases as normal control. Brain tissue samples and tumor samples were collected in the department of pathology. The tissue blocks were retrieved and staining was performed. H&E slides were reproduced for each patient. Patient demographics including age, sex, and tumor histology were recorded.

### 2.2. Digitize Image from H&E Tissue Slide and Pre-Processing of Images

#### 2.2.1. Digitize the H&E Tissue Slide from TCGA-GBM

The original TCGA-GBM dataset was composed of 510 hematoxylin and eosin (H&E) stained histological slides from 262 patients in .svs format. The software Aperio ImageScope version. 12.4.3.5008 was used to view the .svs files. Within the software, the 10× objective was chosen with a total magnification of 100× used to capture the slides one by one with no overlap. The images were saved as .tif format, with 1304 × 839 pixel.

#### 2.2.2. Digitize the H&E Tissue Slide from Local Hospital

The hematoxylin and eosin (H&E)-stained histological slides from GBM patients were placed onto the stage of a light microscope (Zeiss Primostar 3 microscope, Carl Zeiss Microscope GmbH, Jena, Germany). The tissue slide was focused under 10× objective (Zeiss iPlan-Achromat 10× lens) with a total magnification of 100×. Images of pixel 1304 x 839 (same as the image size of TCGA-GBM) were captured one by one with no overlap using the high-resolution CCD camera. (The microscope was equipped with the integrated 4K microscope camera, with Sony CMOS image color sensor, maximum pixel count 3840 (H) × 2160 (W) = 8.3-megapixel HD (4K) and the imaging software (Labscope v2.9). The image was saved as .tif format for further processing.

#### 2.2.3. Standardization and Normalization of Images

A trained medical technologist contoured the regions of interest (ROIs). The ROI of the diseased group is where the cancer cells are located, while the ROI of the control group is where normal brain cells are located. A pathologist checked and confirmed the ROI was accurately contoured. Images were taken within the ROI only.

**Figure 1:**
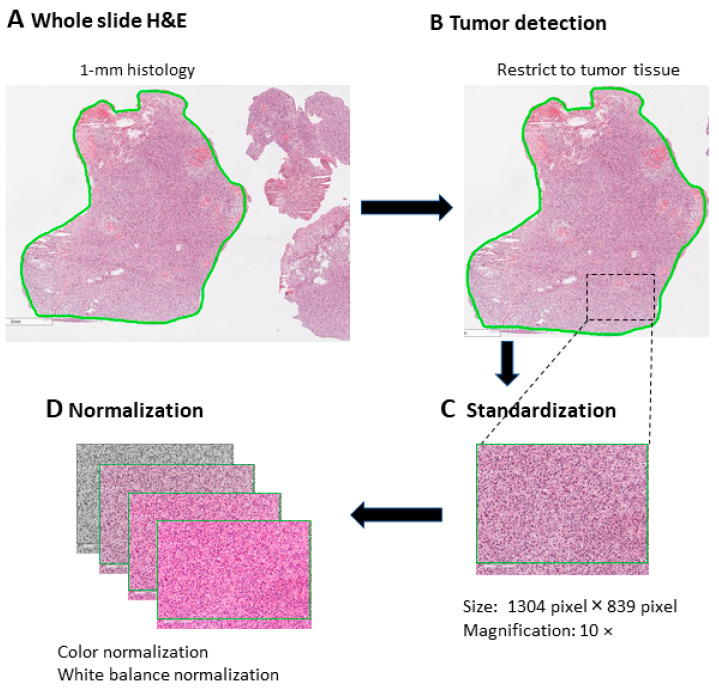
The workflow of digitized image and pre-processing image.

Color staining and white balance normalization were performed for all images collected to ensure the image features collected were standardized. The pre-processing procedures were done to minimize the variation among images collected from various hospitals. The captured images were analyzed for further image feature analysis. The workflow of the digitized image and pre-processing image are presented in Figure 1. Details of the study dataset are presented in Table 1.

### 2.3. Image Feature Extraction from Tissue Slide Images

Twenty-two gray level co-occurrence matrix (GLCM) image features and eleven gray level run length matrix (GLRLM) image features were extracted from the H&E slide images. Image feature extraction was performed by an in-house developed program, built based on the image processing toolbox of MATLAB^®^ (The MathWorks Inc., Natick, Massachusetts, United States) 2021a platform. The definition of 22 GLCM and 11 GLRLM image features are listed in Table 2. The mathematical formula to calculate each feature from the digital image were listed in the File S1.

### 2.4. Study Workflow

The study comprised two parts. Part 1 aimed to build the models for GBM diagnosis using five commonly used machine learning algorithms. In this part, the images from the TCGA-GBM dataset and images from the normal cohort collected from the local hospital were used. A balanced sample was used to build the model, i.e., the number of images was similar in both the GBM and normal group. This was to ensure the model trained was not biased towards one group. In other words, the model did not favor any of the group due to it containing more data [36].

After image pre-processing, all images (from both the GBM and the normal) were randomly split into three independent groups, with 70% for the training group, 15% for the validation group, and 15% for the testing group. Details are shown in Table 3. The images were sent to the in-house developed software for 22 GLCM and 11 GLRLM image features extraction, and then to the classification learner application in Matlab 2021a for model building. The decision tree (DT) algorithm, the extreme boost (EB) algorithm, the random forest (RF) algorithm, the support vector machine (SVM) algorithm, and the linear model algorithm (LM) were employed for model building. Each model was trained, tested, and validated. As a result, five models were built, including DT model, EB model, SVM model, RF model, and LM model.

**Figure 2:**
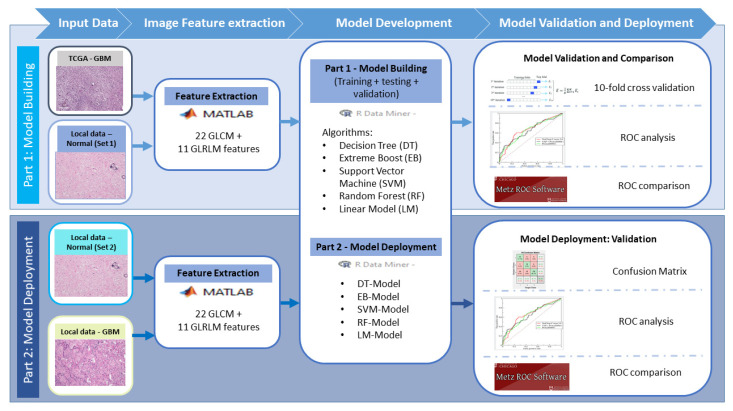
Detail study workflow.

In part 2, independent histopathological images from the local hospital were used for model deployment. The 702 images from local GBM patients and 670 images from the local normal cohort (which were independent from those in the normal cohort for part 1) were used. After image pre-processing, images were sent to the in-house developed software for 22 GLCM and 11 GLRLM image features extraction. This time, the image features were sent to the five models built in part 1 to verify whether the model was able to diagnose GBM from normal. A confusion matrix, ROC analysis, and ROC comparison were conducted to identify the best model for GBM diagnosis. Figure 2 is an illustration diagram of the study workflow.

### 2.5. Machine Learning Algorithms

Five machine-learning algorithms in the classification learner application of Matlab 2021a were employed. They were the decision tree (DT) algorithm, the extreme boost (EB) algorithm, the random forest (RF) algorithm, the support vector machine (SVM) algorithm, and the linear model (LM). These are common machine learning algorithms, which facilitate the comparison of our results with other studies.

For each learning algorithm, the 22 GLCM and 11 GLRLM image features were used. The diagnostic outcome was presented as a binary output, i.e., decimal value ranging between 0 and 1 (with 0 and 1 inclusive). A decimal value of less than 0.5 indicated the model predicting the patient was normal, whereas a decimal value equal to or greater than 0.5 indicated the model predicting the patient suffered from GBM.

### 2.6. Ten-Fold Cross Validation to Minimize Overfitting

To minimize the overfitting and improve the generalization capability of the developed system, a 10-fold cross validation was employed. The dataset was randomly divided into ten groups with an equal number of samples. The first training used the initial nine groups as training data, and the remaining group as testing data. The second training continued with another nine groups as training data and the rest as testing data. The process continued 10 times. The performance of the model was the average of the result computed in these 10 training iterations [37].

### 2.7. Data Analysis

The performance of the 5 machine-learning algorithms were assessed by the overall accuracy, sensitivity, and specificity, as well as the area under the receiver operating characteristic (ROC) curve. Also, a bivariate Chi-square test was performed to calculate the statistical significance of the difference between the ROC curves estimates, using the ROCKIT ver1.1B2 software, developed by the University of Chicago.

### 2.8. Deployment of Models—Verified by Local Data

The models built in Section 2.4 were deployed. The local dataset was used to test the performance of the deployed models. The performance of the five models built in part 1 was assessed by the overall accuracy, sensitivity, and specificity, as well as the area under the receiver operating characteristic (ROC) curve. A bivariate Chi-square test was performed to calculate the statistical significance of the difference between the ROC curves estimates, using the ROCKIT ver1.1B2 software, developed by the University of Chicago.

## 3. Results

### 3.1. Dataset Demographics

**Figure 3:**
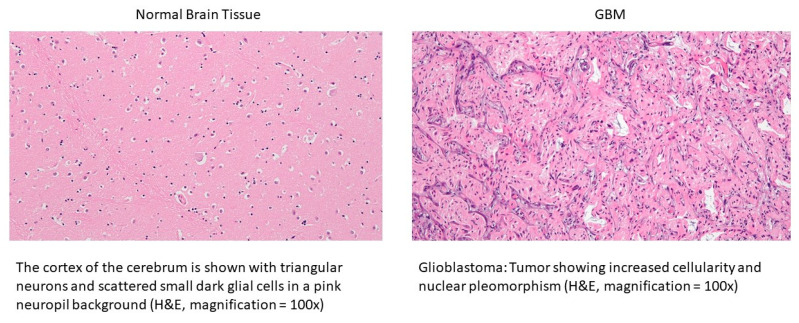
A sample set of the H&E histology of normal brain tissue and GBM with histological characteristics.

The TCGA GBM dataset and the local hospital dataset comprise 262 and 60 patients, respectively. The demographics of the study cohorts are shown in Table 4. A sample set of the H&E histology of normal brain tissue and GBM with histological characteristics is shown in Figure 3.

### 3.2. Image Features Selection

Among the 22 GLCM and 11 GLRLM image features, 15 features separate normal and GBM well. In GLCM, there are 12 image features including contrast (contr), difference variance, dissimilarity (dissi), energy (Energ), entropy (Entro), homogeneity 1 (Homom), homogeneity 2 (homop), maximum probability, sum average, sum entropy, difference entropy, information measure of correlation 1, inverse difference normalized, and inverse difference moment normalized. In GLRLM, there are three image features including gray level non-uniformity, low gray level fun emphasis, and long run low gray level emphasis. Details of each feature are listed in Table 5.

#### 3.2.1. GLCM Image Features

**Figure 4:**
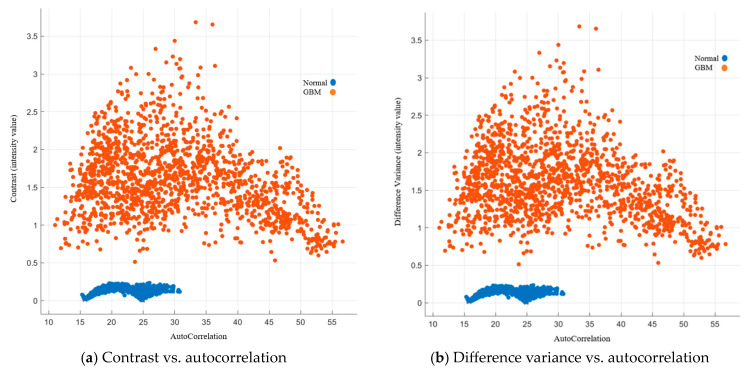
GLCM image features related to local intensity variation.

**Figure 5:**
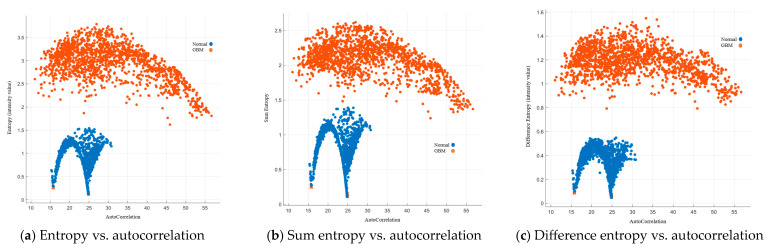
GLCM image features related to entropy.

**Figure 6:**
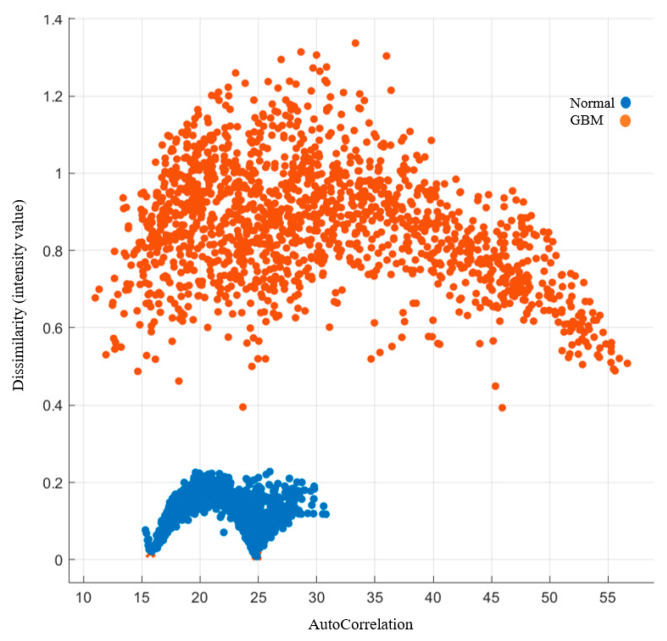
GLCM image features related to dissimilarity.

**Figure 7:**
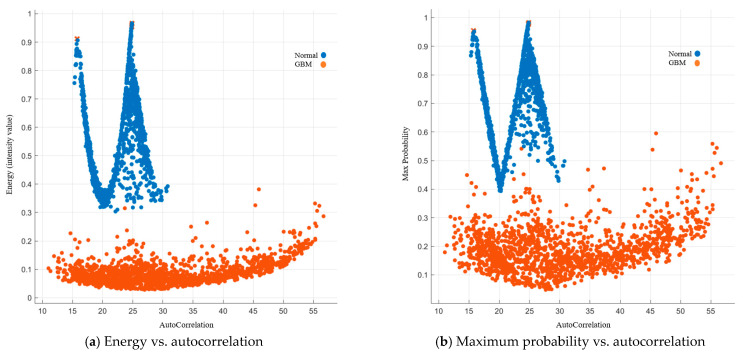
GLCM image features related to energy and maximum probability.

**Figure 8:**
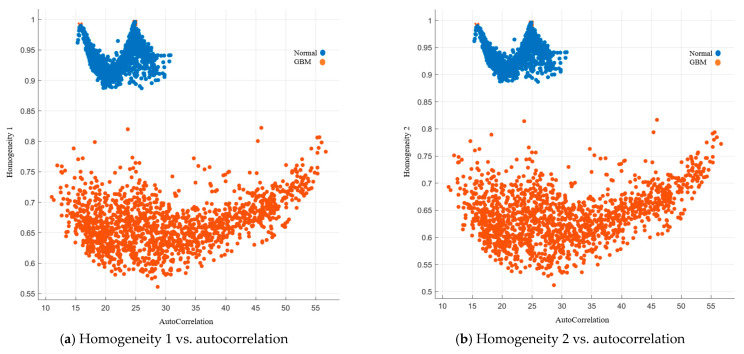

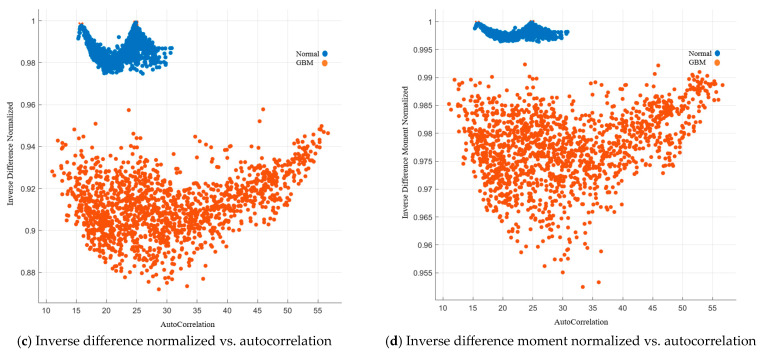
GLCM image features related to homogeneity.

Autocorrelation is a measure of the texture magnitude related to fineness and coarseness. The smaller the number, the finer the image. Moreover, the smaller range of autocorrelation indicated that the image composes of finer details. Our results showed that the range for the normal group was 15–31, while the range for the GBM group was 10.5–57. To present the image features with standardized autocorrelation, all other image features were illustrated versus autocorrelation in Figure 4, Figure 5, Figure 6, Figure 7 and Figure 8.

##### Image Features Related to Local Intensity Variation

Contrast and difference variance are image features that are related to local intensity variation. Contrast in GLCM indicated the intensity variation between adjacent pixels, where a high degree of contrast meant the intensity varied rapidly. The contrast was low for the normal group (blue), which was below 0.4. The contrast ranged from 0.5 to 3.5 for the GBM group. There was a similar situation for difference variance. The details are listed in Figure 4.

##### Image Features Related to Entropy

Entropy specifies the randomness in the image, where the higher value indicates that the image is more complex with higher randomness [33]. Our results showed that the entropy, sum entropy, and difference entropy were below 1.52, 1.3, and 0.6 for the normal group, and above 1.6, 1.5, and 0.8 for the GBM group, respectively. Details are listed in Figure 5.

##### Image Features Related to Dissimilarity

Dissimilarity measures the relationship between the occurrences of pairs with differing intensity values and that of the similar intensity values. The dissimilarity in the normal group was below 0.3 whereas it was higher than 0.3 in the GBM group. The higher the dissimilarity, the higher the difference in average of the image value. Details are listed in Figure 6.

##### Image Features Related to Energy and Maximum Probability

Energy measures the amount of variation within a fixed size window by using local masks. The level, edge, spot, and ripple can be segmented within the image [38]. The energy in the normal group was higher than 0.28 whereas it was lower than 0.28 in the GBM group. A higher energy indicates more instances of intensity value pairs that were next to its neighbor at higher frequencies. Similarly, maximum probability measured the occurrences of the most predominant pair of neighboring intensity value. Our results showed that it was lower than 0.43 in the GBM group whereas it was higher than 0.5 in the normal group. Details are listed in Figure 7.

##### Image Features Related to Homogeneity

Homogeneity 1, also known as inverse difference, is a measure of the uniformity of intensity values of pixel pairs [35]. The higher the value, the more uniform the image intensity. The homogeneity 1 for the normal group was higher than 0.85, while it was lower than 0.85 for the GBM group. Homogeneity 2, also known as inverse difference moment, is a measure of local homogeneity within the image. The weighting is the inverse of the contrast weighting in GLCM. Our results showed that the homogeneity 2 for the normal group was higher than 0.85, while it was lower than 0.85 for the GBM group.

The inverse difference normalized (IDN) and the inverse difference moment normalized (IDMN) are the normalized version of homogeneity 1 and homogeneity 2, respectively. IDN normalized the difference of intensity values between the neighboring pixels by dividing over the total number of discrete intensity values. Our results showed that it was higher than 0.97 and lower than 0.97 for the normal group and GBM group, respectively. For IDMN, it normalizes the square of the difference of intensity values between neighboring pixels by dividing over the square of the total number of discrete intensity values. The cutoff value is 0.995. The GBM group was lower than the cutoff value, while the normal group was higher than the cutoff value. Details are listed in Figure 8.

#### 3.2.2. GLRLM Image Features

GLRLM was first introduced by Galloway et al., 1975 [11]; it assesses the length of a consecutive sequence of pixels with the same grey level. Gray level non-uniformity (GLN) measures the similarity of gray level intensity in the image. The lower the GLN, the more similar the intensity values. Our results showed that within the normal group, the majority of GLN values were lower than 1 × 10^5^, while they were higher than 1 × 10^5^ for the GBM group.

Low gray level run emphasis measures the low gray-level values distribution, a higher value indicating more low gray-level values in the image. Our results showed that LGRE was under 0.3 in the normal group, while it was higher than 0.49 for the GBM group. It indicated that the GBM group image showed a greater concentration of low gray level values in the image.

**Figure 9:**
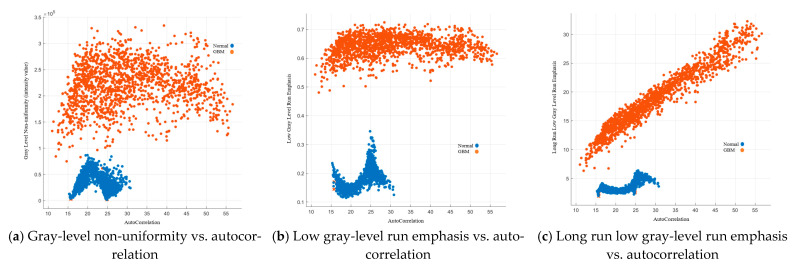
GLRLM image features.

Long run low gray-level emphasis (LRLGE) measures the joint distribution of long run lengths with lower gray-level values. The higher value indicates a longer consecutive sequence of low gray-level values in the image. Our results showed that LRLGE was under 5.1 in the normal group, while it is ranged from 5.2 to 35 for the GBM group. Details are listed in Figure 9.

### 3.3. Model Building and Validation Using Images from TCGA-GBM

**Figure 10:**
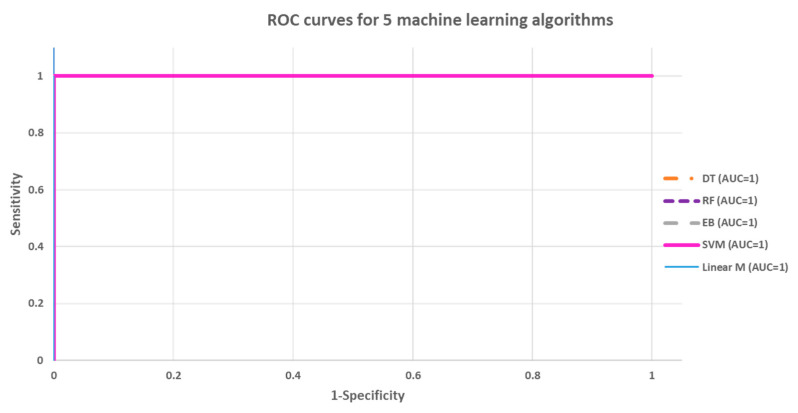
Model building: ROC curve analysis between the five models.

The GLCM and GLRLM image features were extracted from 1500 images from TGBA-GBM patients and 1500 images from normal patients; all five models created achieved excellence performance with 100% accuracy, with 100% sensitivity and specificity. The same results were attained from a 10-fold cross validation. There was no significant difference in the accuracy among the five algorithms of the ROC curves (*p* > 0.05) in the Chi-square test. Detailed results, the ROC curves, and the confusion matrix are shown in Table 5 and Figure 10, respectively.

### 3.4. Model Deployment Using Images from Local Hospital

**Figure 11:**
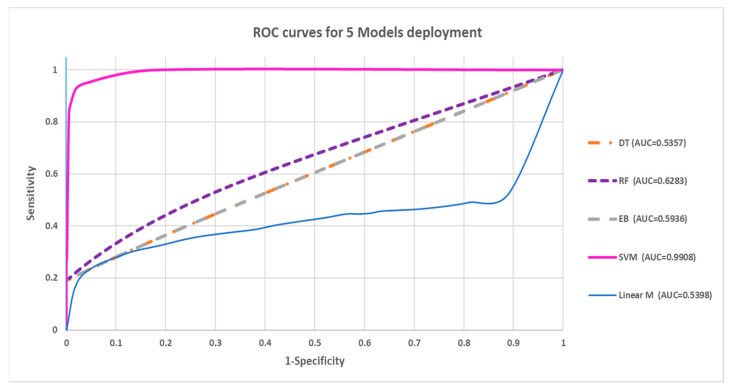
ROC curves for deployment of AI models.

Among the five models, the SVM model showed the highest overall accuracy of 93.5%, with the highest sensitivity of 86.95% and specificity of 99.73%. The other four models showed unsatisfactory results in GBM diagnosis, with 55.0–60.3% accuracy. The main issue was poor performance in sensitivity, with 0% to 20.8% only. Furthermore, the accuracy of the SVM model was statistically significantly better than the other models, with *p* < 0.05 in the Chi-square test. Detailed results, the ROC curves, and the confusion matrix are shown in Table 6 and Figure 11, respectively.

## 4. Discussion

### 4.1. Significance of This Project

In this study, we identified 12 GLCM and 3 GLRLM image features from 22 GLCM and 11 GLRLM image features extracted which can aid in the diagnosis of GBM. Among the five models built, the SVM model proposed in this study demonstrated excellent accuracy with very good sensitivity and specificity. It could potentially be used for GBM diagnosis and future clinical application.

### 4.2. The Value of GLCM and GLRLM Image Features in H&E Images

Traditionally, GBM diagnosis mainly focuses on the identification of cancer features from H& E slides, including cellular morphology, multiple nucleoli, and irregular shape of nucleus, i.e., a morphology-based analysis [39]. Accurate diagnosis required pathologists’ years of diagnostic experience, as well as their concentration. The diagnostic process is labor intensive and time consuming. The reproducibility of manual diagnosis is low and subject to inter-observer agreement. The machine-assisted (AI based) morphological analysis may partially overcome these shortcomings. However, due to the high degree of tumor heterogeneity in GBM, large scale morphological analysis remains challenging [7].

In this study, we proposed to use the GLCM and GLRLM, where they are based on the statistical study of pixel intensity distribution of the image [10] for GBM diagnosis. GLCM represents the frequency of a pixel with gray level i present in a spatial location to a gray level j, in 3 directions, including vertical, horizontal, or diagonal [40]. In an oncology diagnostic setting, the targeted lesions usually present with multiple nucleoli and irregular sizes and shapes. It is expected that the gray-level pixel intensity variation is higher, with higher randomness, higher variation in the gray-level of neighboring pixels, and less homogeneity in GBM compared to normal cells, as the GLCM of lesion and the normal are different in nature [41]. From the first part of this study, we identified 12 GLCM that are significantly different between GBM and the normal group. The GLCM features match the characteristics of GBM histopathological images. Within GLCM, the GBM group was high in contrast and difference variance, indicating that the gray-level intensity variation was higher in the GBM group when compared to the normal group. For image feature-related to entropy, GBM showed a higher value which indicates that it is more complex with higher randomness when compared to the normal group. In view of image features related to dissimilarity, which measure the occurrence of pairs of pixels with differ intensity, the GBM group showed a higher difference in average the image value. For the image feature related to energy, which showed the amount of variation within the image, the GBM group showed a lower value in energy and maximum probability, indicating the image is higher in variation in the gray-level of neighbor pixels when compared to the normal. For homogeneity, the GBM group showed lower values, which indicates that it is less homogeneous in gray-level when compared to the normal group.

In addition, we identified three GLRLM features that are suitable for GBM diagnosis. GLRLM represents the gray-level runs, that is, the length in number of consecutive pixels that have the same gray-level value [11]. Most GBMs exhibit nuclear atypia, multiple mitotic figures, and a high degree of nuclear pleomorphism. Within the tumor, central necrosis is a signature of GBM, which are in yellow in histopathological images, with a peripheral grayish tumor mass [42]. The characteristic features match with our results, where the GBM group showed higher GLN values, which indicates less similarity in intensity value; a higher LGRE value indicating greater concentration of low gray-level value and a higher LRLGE value indicating a longer consecutive sequence of low gray-level value.

### 4.3. Advantage of SVM in Classification

SVM is a binary classifier, which used the hyperplane to define a clear decision boundary to separate various data points based on their classes [43]. In this study, SVM attained the best performance among the five most common machine-learning algorithms in the diagnostic model for GBM. SVM was first introduced by Vapnik as one of the classification algorithms focusing on solving inputs with a non-linear relationship [44]. Based on the results of current study, the accuracy of linear model was low, indicating that the relationship between the image features (GLCM and GLRLM) and the diagnosis were not linear. SVM maps the non-linear inputs into the newly generated space, where the inputs can be separated linearly [45]. In general, SVM has excellent generalization capability relative to other algorithms, especially in the case of fewer training samples. The major advantage of SVM is that it can classify data that are not linearly separable. Moreover, in our study, there were only 33 image features. This is particularly suitable for a multivariate algorithm, such as SVM, to simplify the data interpretation and prevent overfitting [46].

### 4.4. Study Limitations and Further Studies

In this study, an independent local cohort of GBM patients was used for model testing. The sample size was relatively small. Further study is suggested using another cohort of patients from different centers with a larger sample size to ensure the model is generalizable.

In future work, we will continue to investigate the multi-class characterization of brain tumors’ histopathological images. Further sub-class recognition may provide a comprehensive theoretical basis for medical technologists and pathologists for accurate diagnosis of brain tumors.

## 5. Conclusions

In this study, there were 12 GLCM and 3 GLRLM texture features identified which were suitable for GBM diagnosis. These features could be used for AI-based histopathological image analysis. Among the AI models developed, SVM offered the highest accuracy of 93.5% and attained 86.95% and 99.73% of sensitivity and specificity, respectively, demonstrating great potential for future clinical application.

## Figures and Tables

**Table 1:** Details of study dataset.

	Cohort 1: for Model Building (TCGA–GBM)		Cohort 2: for Model Deployment (Local Hospital)	
	No of Participants	No of Images	No of Participants	No of Images
GBM	262	1500	60	702
Normal	40	1500	20	670

**Table 2:** GLCM and GLRLM image features.

Gray Level Co-Occurrence Matrix (GLCM)		Gray Level Run Length Matrix (GLRLM) [30,31]
Autocorrelation [32]	Maximum Probability [32]	Short Run Emphasis
Contrast [32,33]	Sum of square [33]	Long Run Emphasis
Correlation 1 [34]	Sum average [33]	Gray Level Non-uniformity
Correlation 2 [32,33]	Sum variance [33]	Run Length Non-uniformity
Cluster prominence [32]	Sum entropy [33]	Run Percentage
Cluster Shade [32]	Difference variance [33]	Low Gray-level Run Emphasis
Dissimilarity [32]	Difference entropy [33]	High Gray-level Run Emphasis
Energy [32,33]	Information measure of correlation 1 [33]	Short Run Low Gray-level Run Emphasis
Entropy [32]	Information measure of correlation 2 [33]	Short Run High Gray-level Run Emphasis
Homogeneity 1 [34]	Inverse Difference normalized [35]	Long Run Low Gray-level Run Emphasis
Homogeneity 2 [32]	Inverse Difference moment normalized [35]	Long Rg equations to obtain each features were listed in the . es were y. Rrun High Gray-level Run Emphasis

**Table 3:** Image distribution of the study for model development (Part 1) and model deployment (Part 2).

**Part 1** **Model** **development**		**GBM group (*n* = 1500)**			**Normal Group (*n* = 1500)**		
	Group	Training	Validation	Testing	Training	Validation	Testing
	Percentage	70%	15%	15%	70%	15%	15%
	Sample size	1050	225	225	1050	225	225
**Part 2** **Model** **deployment**		**GBM group (*n* = 702)**			**Normal Group (*n* = 670)**		
	Group	Training	Validation	Testing	Training	Validation	Testing
	Percentage	70%	15%	15%	70%	15%	15%
	Sample size	492	105	105	470	100	100

**Table 4:** Demographics of the GBM of the TCGA and the local hospital dataset.

	Part 1 For Model Development		Part 2 For Model Deployment	
	GBM	Normal	GBM	Normal
**No of Participants**	262	40	60	20
Age Range Mean ±SD Sex (M:F)	**14–86** 58.96 ± 13.95 159:101	NIL	**33–75** 61 ± 11.06 42:18	NIL

**Table 5:** Overall accuracy, sensitivity, and specificity for GBM diagnosis using H&E image features.

Algorithm	Overall Accuracy	Sensitivity	Specificity	Area under the ROC Curve	Precision	Recall	F1 Score
Decision Tree (DT)	100%	100%	100%	1	100%	100%	1
Extreme Boost (EB)	100%	100%	100%	1	100%	100%	1
Random Forest (RF)	100%	100%	100%	1	100%	100%	1
Support Vector Machine (SVM)	100%	100%	100%	1	100%	100%	1
Linear Model (LM)	100%	100%	100%	1	100%	100%	1

**Table 6:** Overall accuracy, sensitivity, and specificity in model deployment.

Algorithm	Overall Accuracy	Sensitivity	Specificity	Area under the ROC Curve	Precision	Recall	F1 Score
DT-Model	59.4%	18.82%	1	0.5357	17.2%	100%	29.3%
EB-Model	59.4%	18.82	1	0.5936	17.2%	100%	29.3%
RF-Model	60.3%	21.06%	1	0.6283	20.8%	100%	34.4%
SVM-Model	93.5%	86.95%	99.73%	0.9908	82.9%	99.7%	90.5%
LM-Model	55.0%	11.80%	1	0.5398	0%	100%	0%

## Data Availability

Publicly available datasets were used in this study. The dataset can be obtained at following website: https://wiki.cancerimagingarchive.net/pages/viewpage.action?pageId=1966258 (accessed on 22 January 2023). Data used in preparation of this article were obtained from the Cancer Imaging Archive (TCIA) database (https://wiki.cancerimagingarchive.net/display/Public/Wiki) (accessed on 22 January 2023). The local dataset will be available upon request.

## Supplementary Materials

The following supporting information can be downloaded at: https://www.mdpi.com/article/10.3390/cancers15205063/s1, File S1: GLCM & GLRLM equations.
