# Supplementary material for: AI Deployment on GBM Diagnosis: A Novel Approach to Analyze Histopathological Images Using Image Feature-Based Analysis
Source: Cancers (Basel). 2023 Oct 19;15(20):5063. doi: 10.3390/cancers15205063 (PMC10605241; doi:10.3390/cancers15205063)
Supplement: Supplementary file 1 [file cancers-15-05063-s001.zip › cancers-2643432-supplementary.pdf]

File S1 GLCM & GLRLM equations

GLCM equations

$$\text{autocorrelation} = \sum_{i=1}^{N_g} \sum_{j=1}^{N_g} p(i,j)ij$$

$$\text{contrast} = \sum_{i=1}^{N_g} \sum_{j=1}^{N_g} (i-j)^2 p(i,j)$$

$$\text{correlation} = \frac{\sum_{i=1}^{N_g} \sum_{j=1}^{N_g} p(i,j)ij - \mu_x \mu_y}{\sigma_x(i)\sigma_y(j)}$$

$$\text{MCC} = \sqrt{\text{second largest eigenvalue of Q}}$$

$$Q(i,j) = \sum_{k=0}^{N_g} \frac{p(i,k)p(j,k)}{p_x(i)p_y(k)}$$

$$\text{cluster prominence} = \sum_{i=1}^{N_g} \sum_{j=1}^{N_g} (i+j-\mu_x-\mu_y)^4 p(i,j)$$

$$\text{cluster shade} = \sum_{i=1}^{N_g} \sum_{j=1}^{N_g} (i+j-\mu_x-\mu_y)^3 p(i,j)$$

$$\text{dissimilarity} = \sum_{i=1}^{N_g} \sum_{j=1}^{N_g} |i-j| p(i,j)$$

$$\text{energy} = \sum_{i=1}^{N_p} (\mathrm{X}(i) + c)^2$$

$$\text{entropy} = -\sum_{i=1}^{N_g} p(i) \log_2 (p(i) + \epsilon)$$

$$\text{homogeneity 1} = \sum_{i=1}^{N_g} \sum_{j=1}^{N_g} \frac{p(i,j)}{1 + |i - j|}$$

$$\text{homogeneity 2} = \sum_{i=1}^{N_g} \sum_{j=1}^{N_g} \frac{p(i,j)}{1 + |i - j|^2}$$

$$\text{maximum probability} = max(p(i,j))$$

$$\text{sum squares} = \sum_{i=1}^{N_g} \sum_{j=1}^{N_g} (i - \mu_x)^2 p(i,j)$$

$$\text{sum average} = \sum_{k=2}^{2N_g} p_{x+y}(k) k$$

$$\text{sum variance} = \sum_{k=2}^{2N_g} (k - SA)^2 p_{x+y}(k)$$

$$\text{sum entropy} = \sum_{k=2}^{2N_g} p_{x+y}(k) \log_2 (p_{x+y}(k) + \epsilon)$$

$$\text{difference variance} = \sum_{k=0}^{N_g-1} (k - DA)^2 p_{x-y}(k)$$

$$\text{difference entropy} = \sum_{k=0}^{N_g-1} p_{x-y}(k) \log_2 \left(p_{x-y}(k) + \epsilon\right)$$

$$\text{IMC 1} = \frac{HXY - HXY1}{max\{HX, HY\}}$$

$$\text{IMC 2} = \sqrt{1 - e^{-2(HXY2 - HXY)}}$$

$$\text{IDN} = \sum_{k=0}^{N_g-1} \frac{p_{x-y}(k)}{1 + (\frac{k}{N_g})}$$

$$\text{IDMN} = \sum_{k=0}^{N_g-1} \frac{p_{x-y}(k)}{1 + (\frac{k^2}{N_g^2})}$$

GLRLM equations

$$\mathrm{SRE} = \frac{\sum_{i=1}^{N_g} \sum_{j=1}^{N_r} \frac{\mathrm{P}(i,j|\theta)}{j^2}}{N_r(\theta)}$$

$$\mathrm{LRE} = \frac{\sum_{i=1}^{N_g} \sum_{j=1}^{N_r} \mathrm{P}(i,j|\theta) j^2}{N_r(\theta)}$$

$$\mathrm{GLN} = \frac{\sum_{i=1}^{N_g} \left(\sum_{j=1}^{N_r} \mathrm{P}(i,j|\theta)\right)^2}{N_r(\theta)}$$

$$\mathrm{RLN} = \frac{\sum_{j=1}^{N_r} \left(\sum_{i=1}^{N_g} \mathrm{P}(i,j|\theta)\right)^2}{N_r(\theta)}$$

$$\mathrm{RP} = \frac{N_r(\theta)}{N_p}$$

$$\mathrm{LGLRE} = \frac{\sum_{i=1}^{N_g} \sum_{j=1}^{N_r} \frac{\mathrm{P}(i,j|\theta)}{i^2}}{N_r(\theta)}$$

$$\mathrm{HGLRE} = \frac{\sum_{i=1}^{N_g} \sum_{j=1}^{N_r} \mathrm{P}(i,j|\theta) i^2}{N_r(\theta)}$$

$$\mathrm{SRLGLE} = \frac{\sum_{i=1}^{N_g} \sum_{j=1}^{N_r} \frac{\mathrm{P}(i,j|\theta)}{i^2 j^2}}{N_r(\theta)}$$

$$\mathrm{SRHGLE} = \frac{\sum_{i=1}^{N_g} \sum_{j=1}^{N_r} \frac{\mathrm{P}(i,j|\theta) i^2}{j^2}}{N_r(\theta)}$$

$$\mathrm{LRLGLRE} = \frac{\sum_{i=1}^{N_g} \sum_{j=1}^{N_r} \frac{\mathrm{P}(i,j|\theta) j^2}{i^2}}{N_r(\theta)}$$

$$\mathrm{LRHGLRE} = \frac{\sum_{i=1}^{N_g} \sum_{j=1}^{N_r} \mathrm{P}(i,j|\theta) i^2 j^2}{N_r(\theta)}$$
